# Volcanogenic Pseudo-Fossils from the ∼3.48 Ga Dresser Formation, Pilbara, Western Australia

**DOI:** 10.1089/ast.2017.1734

**Published:** 2018-05-01

**Authors:** David Wacey, Nora Noffke, Martin Saunders, Paul Guagliardo, David M. Pyle

**Affiliations:** ^1^ Centre for Microscopy Characterisation and Analysis, The University of Western Australia, Perth, Australia.; ^2^ Department of Ocean, Earth and Atmospheric Sciences, Old Dominion University, Norfolk, Virginia, USA.; ^3^ Department of Earth Sciences, University of Oxford, Oxford, UK.

## Abstract

The ∼3.48 billion-year-old Dresser Formation, Pilbara Craton, Western Australia, is a key geological unit for the study of Earth's earliest life and the habitats it occupied. Here, we describe a new suite of spheroidal to lenticular microstructures that morphologically resemble some previously reported Archean microfossils. Correlative microscopy shows that these objects have a size distribution, wall ultrastructure, and chemistry that are incompatible with a microfossil origin and instead are interpreted as pyritized and silicified fragments of vesicular volcanic glass. Organic kerogenous material is associated with much of the altered volcanic glass; variable quantities of organic carbon line or fill the insides of some individual vesicles, while relatively large, tufted organic-rich laminae envelop multiple vesicles.

The microstructures reported herein constitute a new type of abiogenic artifact (pseudo-fossil) that must be considered when evaluating potential signs of early life on Earth or elsewhere. In the sample studied here, where hundreds of these microstructures are present, the combined evidence permits a relatively straightforward interpretation as vesicular volcanic glass. However, reworked, isolated, and silicified microstructures of this type may prove particularly problematic in early or extraterrestrial life studies since they adsorb carbon onto their surfaces and are readily pyritized, mimicking a common preservation mechanism for *bona fide* microfossils. In those cases, nanoscale analysis of wall ultrastructure would be required to firmly exclude a biological origin. Key Words: Microfossils—Pseudo-fossils—Volcanic vesicles—Archean life—Pilbara Craton—Dresser Formation. Astrobiology 18, 539–555.

## 1. Introduction

The two regions on Earth that have played dominant roles in the identification of early Archean microfossils, namely, the Barberton Mountain Land of South Africa and the Pilbara Craton of Western Australia, are dominated by volcanic sequences, and both contain a significant component of volcanoclastic sediments (Walsh, 2004; Van Kranendonk, 2006; Westall *et al.,* 2006; Van Kranendonk *et al.,* 2007). Chert and sandstone units from which microfossils have been described (*e.g.,* Westall *et al.,* 2006; Sugitani *et al.,* 2007, 2010; Wacey *et al.,* 2011) frequently occur intercalated with such volcanoclastic sediment, and some microfossil-hosting cherts have been interpreted as silicified volcanoclastic sediment (Lowe, 1999; Westall *et al.,* 2006). Hence, vesicular volcanic glass and microbial textures could occur in close proximity to one another, within the same rock beds or even the same thin sections.

The recognition of the similarity between vesicular volcanic microstructures and potential Archean cells dates back at least half a century (Engel *et al.,* 1968). However, such early studies were concerned only with differentiating vesicles in basalt versus potential microfossils in chert, two very distinct lithologies that can easily be differentiated in the field or in thin section; early studies did not consider that altered vesicular volcanic glass could be reworked into chert or other sediments. More recent work has shown that vesicular volcanics can occur as microclasts within Archean bedded chert (Westall *et al.,* 2006; Brasier *et al.,* 2013) or silicified sandstone (Wacey *et al.,* 2017). These are typically heavily silicified, replacing and disguising the original volcanic composition (*e.g.,* Westall *et al.,* 2006); some Archean vesicular clasts have been shown to contain linings/coatings of organic material (Lepot *et al.,* 2011; Brasier *et al.,* 2013), while others appear to have been colonized by biofilms (Westall *et al.,* 2006, 2011).

The morphological similarity between organic-lined volcanic vesicles and early biotic cells, where both have been silicified, raises problems for robustly identifying early life on Earth or elsewhere. It is notable that, in discussions concerning the biogenicity of putative Archean cells, an origin as altered vesicular volcanic glass has, to the best of our knowledge, never been considered. In contrast, other abiogenic mechanisms for creating spheroidal to lenticular microstructures, for example as carbon-coated silica spherulites, have been routinely discussed (*e.g.,* Ueno *et al.,* 2006; Westall *et al.,* 2006; Sugitani *et al.,* 2007). This contribution aims to bring microfossil-like volcanic microstructures to the attention of the wider astrobiological community. We illustrate a range of microstructures interpreted as silicified and pyritized vesicular volcanics in one of the most important geological units for the study of early life on Earth, noting their association with kerogenous organic material. We discuss the similarities and differences between these structures and *bona fide* cells, and between these structures and some previously described putative Archean microfossils.

## 2. Materials and Methods

### 2.1. Field location/sampling

**FIG. 1.**
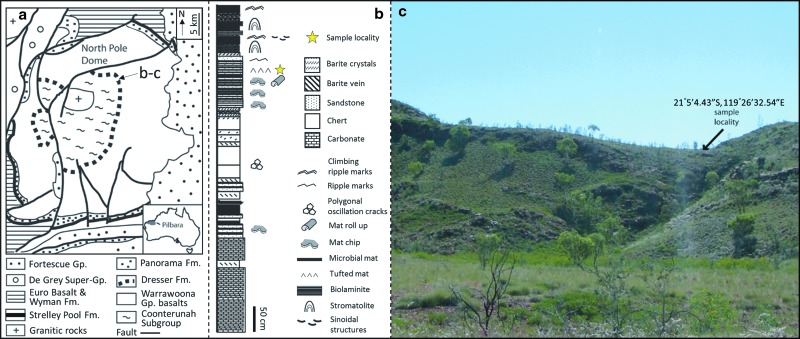
Sample location. (**a**) Geological sketch map showing the distribution of the ∼3.48 Ga Dresser Formation in the North Pole Dome, East Pilbara Terrane, Pilbara Craton, Western Australia (modified from Van Kranendonk *et al.,* 2008). (**b**) Stratigraphic log of the Dresser Formation at Stromatolite Hill showing the distribution of putative biosedimentary structures and the position of the study sample (modified from Wacey *et al.,* 2015). (**c**) Field photograph of Stromatolite Hill showing position and GPS coordinates for the study sample.

The Dresser Formation is located within the East Pilbara granite greenstone terrane of the Pilbara Craton, Western Australia (Fig. 1a). The formation contains some of Earth's oldest and best-preserved volcanic and sedimentary rocks (Fig. 1b; Barley *et al.,* 1979; Hickman, 2012), and its age is well constrained at 3.481 ± 3.5 Ga (Australian Stratigraphic Names Database, Dresser Formation Stratigraphic Number 36957). The Dresser Formation is exposed in a *ca.* 25 km^2^ area in the North Pole Dome and includes bedded chert, carbonate, and siliciclastic portions, plus pillow basalt and dolerite (Nijman *et al.,* 1999; Van Kranendonk *et al.,* 2008). The sedimentary rocks are interpreted to have been deposited under shallow water and low-energy conditions (Buick and Dunlop, 1990) within a caldera-like setting, with significant contemporaneous growth faulting and tectonic activity accompanied by volcanic activity and hydrothermal fluid flow (Van Kranendonk *et al.,* 2008; Djokic *et al.,* 2017). The sample (*ca.* 17 × 9 × 7 cm in size) was collected from an outcrop on the slope of “Stromatolite Hill,” Fig. 1c (Noffke *et al.,* 2013), and thin sections were made from the central nonweathered portion.

### 2.2. Optical microscopy

Petrographic analysis was carried out on four standard uncovered polished geological thin sections (∼30 μm thick) by using a Leica DM2500M microscope, with 4× , 10× , 20× , and 50× lenses, located within the Centre for Microscopy Characterisation and Analysis (CMCA) at The University of Western Australia (UWA). Images were captured with a digital camera and Toupview imaging software.

### 2.3. Laser Raman microspectroscopy

Laser Raman analyses were carried out at the University of Bergen with a Horiba LabRAM HR800 integrated confocal Raman system and LabSpec5 acquisition and analysis software, and at UWA with a WITec alpha 300RA+ instrument and WITec Project/Control FOUR acquisition and analysis software. Samples were standard uncovered geological thin sections, which allowed optical and chemical maps to be superimposed. At Bergen, analyses were carried out by using a 514.5 nm laser, 100 μm confocal hole, 1800 l/mm grating and 50 × /0.5 objective lens, while at UWA analyses were carried out by using a 785 nm laser, 100 μm confocal hole, 600 l/mm grating, plus 20 × /0.4 and 100 × /0.9 objective lenses. Laser centering and spectral calibration were performed daily on a silicon chip with characteristic Si Raman band of 520.4 cm^−1^. Count rates were optimized prior to point spectra acquisition or hyperspectral mapping by using the dominant quartz Raman band of 465 cm^−1^. The laser was focused at least 1 μm below the surface of the thin sections to avoid surface polishing effects, and the laser excitation intensity at the sample surface was in the 1–5 mW range. Spectra were collected in the 100–1800 rel. cm^−1^ region in order that both 1^st^-order mineral vibration modes and 1^st^-order carbonaceous vibration modes could be examined simultaneously. For mineral identification from Raman spectra, dual acquisitions were taken from each analysis point, each with an acquisition time of 4 s. Spectral decomposition and subsequent image processing were performed with the Labspec5 or Project FOUR software, with baseline subtraction using a 3^rd^-, 4^th^-, or 5^th^-order polynomial. Raman maps were acquired with the spectral center of the detector adjusted to 944 cm^−1^ and with 1–1.5 μm spatial resolution. Carbon maps were created by integrating over the ∼1600 cm^−1^ “G” Raman band, quartz maps by using the ∼465 cm^−1^ Raman quartz band, pyrite maps the ∼378 cm^−1^ Raman pyrite band, and anatase maps the ∼145 cm^−1^ Raman anatase band. Control checks for contamination by epoxy were carried out by using the ∼830 cm^−1^ and 650 cm^−1^ Raman bands (see Supplementary Figs. S1 and S2; Supplementary Data are available online at www.liebertonline.com/ast). Three-color overlay images were created with Image J software.

### 2.4. Focused ion beam (FIB) preparation of transmission electron microscope (TEM) samples

A dual-beam FIB system (FEI Nova NanoLab) at the Electron Microscopy Unit, University of New South Wales, was used to prepare TEM wafers from the thin sections described above, coated with ∼30 nm of gold. Electron beam imaging within the dual-beam FIB was used to identify microstructures of interest in the thin sections, allowing site-specific TEM samples to be prepared. The TEM sections were prepared by a series of steps involving different ion beam energies and currents (see Wacey *et al.,* 2012, for details), resulting in ultrathin wafers of *c.* 100 nm thickness. These TEM wafers were extracted with an *ex situ* micromanipulator and deposited on continuous-carbon copper TEM grids. FIB preparation of TEM sections allows features below the surface of the thin sections to be targeted, thus eliminating the risk of surface contamination producing artifacts.

### 2.5. TEM analysis of FIB-milled wafers

Transmission electron microscope data were obtained with a JEOL 2100 LaB6 TEM equipped with a Gatan Orius CCD camera and Tridiem energy filter operating at 200 kV, located in CMCA. Energy-filtered TEM elemental maps were obtained by using the conventional three-window technique (Brydson, 2001), with energy windows selected to provide optimum signal-to-noise.

### 2.6. FIB-SEM 3D nanotomography

Focused ion beam milling and scanning electron microscope (SEM) imaging in three dimensions was performed on a FEI Helios Plasma dual-beam instrument at the Hillsboro NanoPort of Thermo Fisher Scientific. The protocol was a significantly modified version of that described by Wacey *et al.* (2012), with milling and imaging parameters optimized to suit the type of sample (*i.e.,* a relatively large pyritic microstructure within a silica matrix). A region of interest (ROI) previously identified by optical microscopy, approximately 140 × 140 × 30 μm in size, was covered with a protective (*c.* 2 μm thick) platinum layer. Bulk milling to isolate the large ROI was performed with a Xe plasma ion beam; the ROI was then extracted from the thin section with a micromanipulator and attached to a carrier grid to perform the 3D analysis. Three-dimensional milling and imaging was performed with the Auto Slice & View G4 software with a 59 nA beam current and slice thickness of 100 nm. Each newly milled face was imaged (6060 × 2578 pixels, resulting in ∼23 nm pixel size) by using a concentric backscatter detector (CBS) and SEM parameters of 2 kV accelerating voltage and 3.2 nA beam current. A total of 1383 slices was milled, giving a total analysis volume of approximately 580,000 μm^3^. The sequential FIB-SEM nanotomography images were imported into the AVIZO 8.0 software package. Here, they were stacked, aligned, and individual components (*e.g.,* pyrite, which is bright white on the CBS images) were segmented. Models were visualized and rendered in AVIZO 8.0, and images were captured from multiple orientations in 3D space.

### 2.7. Nanoscale secondary ion mass spectrometry (NanoSIMS) ion mapping

High spatial resolution and high sensitivity ion mapping was performed with a CAMECA NanoSIMS 50, located at CMCA. The sample mount was the same as that used during sulfur isotope analysis by Wacey *et al.* (2015), having subsequently been repolished and coated with a thin (*c.* 10 nm) layer of gold to provide conductivity at high voltage. Potential contamination of surface pores by epoxy was checked for using Raman micro-spectroscopy (Supplementary Figs. S1 and S2), and these areas were avoided during NanoSIMS analyses. Details of qualitative ion mapping with NanoSIMS in multicollector mode were given by Wacey *et al.* (2008) and Kilburn and Wacey (2011). Briefly, a focused primary Cs^+^ ion beam, with a beam current of 2–4 pA, was rastered over the sample surface, and the sputtered ions were extracted to a double focusing mass spectrometer. Images with sub-100 nm spatial resolution mapping relative ion intensity were acquired over fields of view ranging from 25 to 40 μm^2^. Prior to each analysis, the sample area was pre-sputtered to remove surface contamination, implant Cs^+^ ions into the sample matrix, and attain an approximate steady state of secondary ion emission (*cf.* Gnaser, 2003). Ion maps of oxygen (^16^O^−^), carbon (^12^C_2_^−^), nitrogen (^12^C^14^N^−^), sulfur (^34^S^−^), and iron sulfide (^56^Fe^32^S^−^) were then produced simultaneously from the same sputtered volumes of sample. Two-color overlay images of ^12^C^14^N^−^ and ^34^S^−^ were created with Image J software. Only relative concentrations of ions can be obtained when using this NanoSIMS methodology. Without multiple standards, no inferences can be made from these data concerning either the absolute concentration of elements or the percentage concentration of one element compared to another.

## 3. Results

### 3.1. Microstructure morphology, distribution, and chemistry

**FIG. 2.**
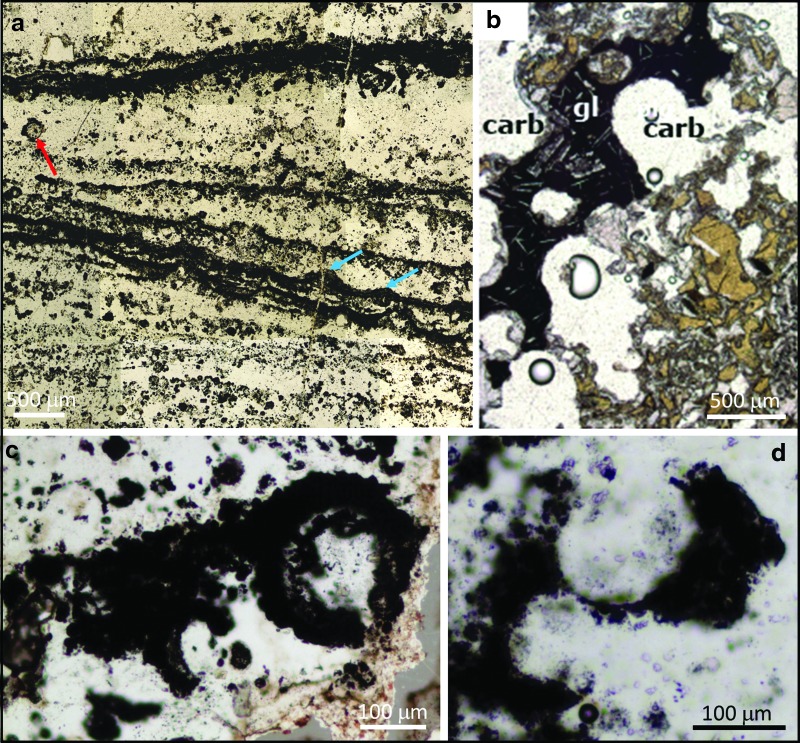
(**a**) Overview of a petrographic thin section of the study sample from the Dresser Formation showing the dominant microtextures, including abundant vesicular structures (one large example marked by red arrow). Note also the dark, sometimes tufted, laminae (blue arrows) that envelop multiple vesicles. (**b**) Photomicrograph from a Miocene volcanoclastic sediment from the South China Sea showing devitrified basaltic volcanic glass (gl) with vesicles now infilled with carbonate (carb); image reproduced with permission from Li *et al.* (2015). (**c**–**d**) Microstructures from the Dresser Formation whose morphologies are analogous to the fragment of Miocene vesicular devitrified volcanic glass in (b).

**FIG. 3.**
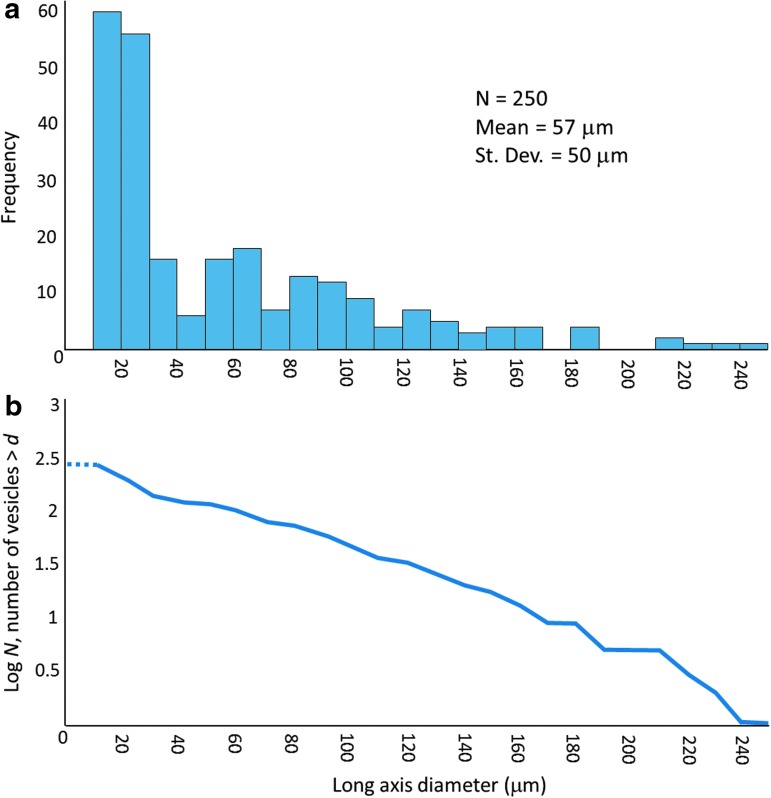
(**a**) Frequency distribution of Dresser Formation vesicle diameters (plotted in such a way as to compare with microfossil frequency distributions given in Schopf, 1976, and Wacey *et al.,* 2011). (**b**) Cumulative distribution of Dresser Formation vesicle diameters (plotted in such a way as to compare with cumulative scoria bubble diameters given in Rust and Cashman, 2011).

**FIG. 4.**
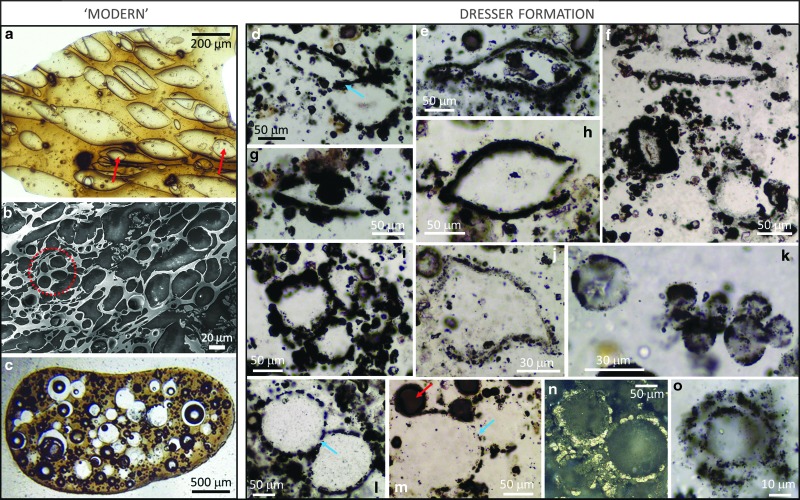
Comparisons of vesicular microstructures in “modern” volcanic glass (a–c) with those in the Dresser Formation (d–o). (**a**) A thin sheet of modern volcanic glass showing a variety of spheroidal to lenticular vesicles; note instances of spheroidal vesicles superimposed on lenticular vesicles (arrowed). (**b**) SEM image of a thin slice of 4 Ma volcanic glass from offshore Montserrat showing a wide range of vesicle shapes and sizes; image reproduced with permission from Jutzeler *et al.* (2017). (**c**) Photomicrograph of volcanic glass from the 1959 Kilauea Iki eruption showing mainly spherical vesicles, some with mineral infill, some empty, with many coalescing; image reproduced with permission from Rutherford and Papale (2009). (**d**–**h**) Examples of lenticular microstructures from the Dresser Formation (compare with (a)); note sphere within lens shown in (g). (**i**) Group of four vesicles from the Dresser Formation (compare with circled vesicles in (b)). (**j**) Slightly deformed vesicle or small glass shard from the Dresser Formation. (**k**) Multiple small vesicles from the Dresser Formation. (**l**–**n**) Examples of spheroidal vesicles with shared walls or interconnection from the Dresser Formation (note coalescence of vesicles in (m) (blue arrow) and compare with (c)). (**o**) Vesicle with alternating layers of silica and pyrite mineral infill from the Dresser Formation.

Vesicular microstructures are abundant in all thin sections examined, occurring through most of the ∼5 cm stratigraphic depth covered by each thin section. They are most abundant in association with ∼50–300 μm thick dark (pyrite and carbon-rich), tufted laminae, but also occur in clearer nonlaminated regions (Fig. 2a). They range in shape from spheroidal to elliptical to eye-shaped and lenticular, with spheroidal forms dominating, especially for the smaller vesicles. They range in long-axis diameter from ∼10 to ∼250 μm. A size frequency distribution (Fig. 3a; *n* = 250) shows that smaller vesicles (10–30 μm) are most common, but both the mean vesicle size (57 μm) and the standard deviation (50 μm) are large. Vesicles can be solitary (Fig. 2a, 4h), but commonly they occur as pairs (Fig. 4d–4e, 4l–4m), triplets (Fig. 4n), and larger groups (Fig. 4i, 4k), occasionally arranged as chains; many share common walls (Fig. 4d, 4l–4m, blue arrows) and show evidence for coalescence (Fig. 4l–4m). Several vesicles are incomplete, with distinctive curved walls being abruptly terminated by silica-filled gaps, giving the impression of erosion or rupturing (Fig. 2c–2d).

**FIG. 5.**
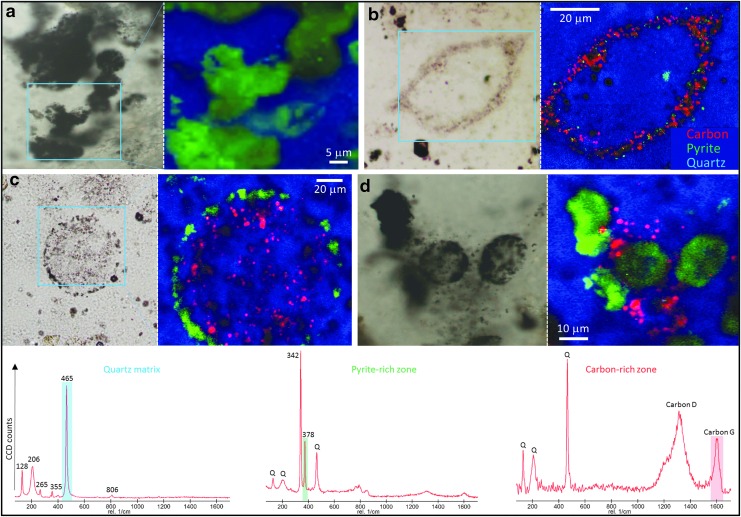
Association of organic material with the Dresser Formation vesicles. (**a**) Photomicrograph and associated Raman phase map showing no organic material associated with a group of small vesicles. (**b**) Photomicrograph and associated Raman phase map showing significant amounts of organic carbon within, and potentially lining, the wall zone of a larger vesicle. (**c**) Photomicrograph and associated Raman phase map showing plentiful organic carbon within a large vesicle but not directly associated with the pyritic “wall.” (**d**) Photomicrograph and associated Raman phase map showing significant organic carbon in the vicinity of a group of small vesicles but not directly correlated with them. For all Raman phase maps, carbon is red, pyrite is green, and quartz is blue. Along the bottom row are representative Raman spectra for the quartz matrix, pyrite-rich zones, and carbon-rich zones. The Raman bands used for mapping each of these phases are shaded (quartz ∼465 cm^−1^ in blue; pyrite ∼378 cm^−1^ in green; and carbon G ∼ 1600 cm^−1^ in red).

**FIG. 6.**
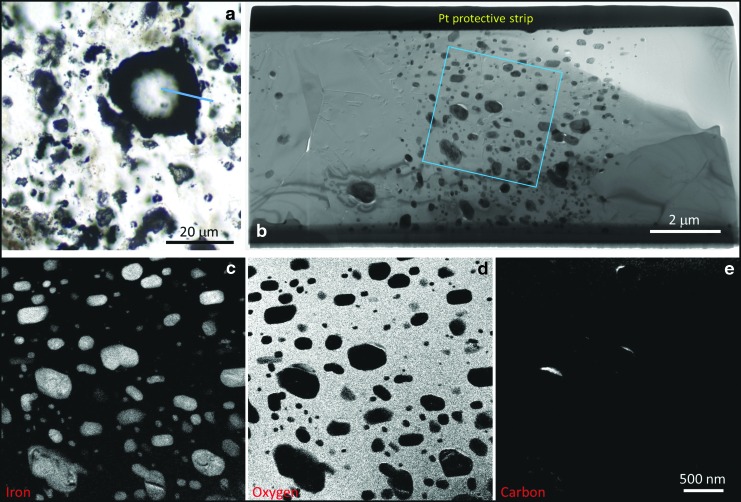
High-resolution analysis of a typical vesicular microstructure from the Dresser Formation. (**a**) Photomicrograph of a spheroidal vesicle; blue line indicates region extracted for TEM analysis. (**b**) Bright-field TEM image showing that the vesicle “wall” comprises silica (mid-gray) with hundreds of nanograins of pyrite (dark gray). (**c**–**e**) Energy-filtered TEM elemental maps of iron, oxygen, and carbon from the boxed area in (b). Small amounts of carbon appear to be associated with only three of the pyrite nanograins.

**FIG. 7.**
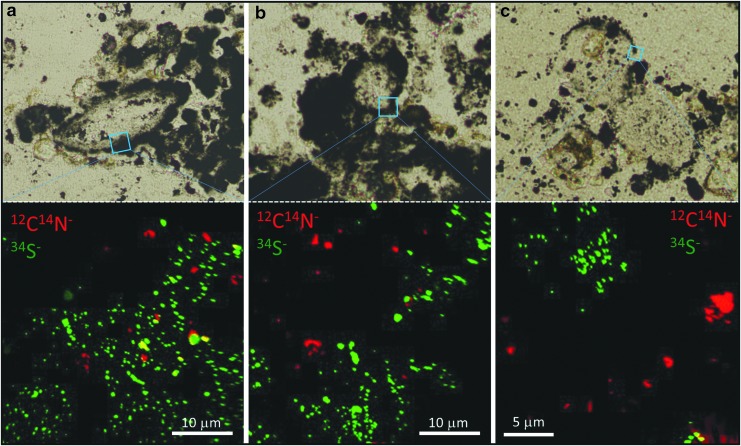
Nanoscale association of organic material and pyritized vesicles. Three sets of photomicrographs and accompanying NanoSIMS ion maps showing that small amounts of organic material occur in close proximity to pyritized vesicle walls but rarely within the walls. For the NanoSIMS maps, organic material is in red, pyrite in green, and quartz in black.

**FIG. 8.**
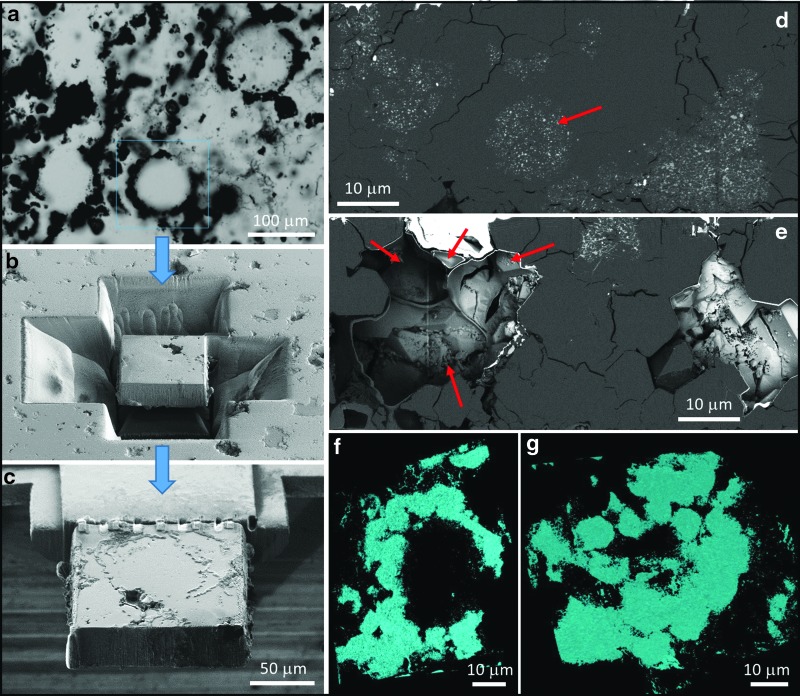
Three-dimensional analysis of a typical vesicular microstructure from the Dresser Formation. (**a**) Photomicrograph of a spheroidal vesicle; blue box indicates the ROI analyzed in 3D. (**b**) SEM image showing large trenches milled around the structure in preparation for lifting out the ROI for 3D analysis. (**c**) SEM image of the ROI attached to the analysis grid prior to 3D data collection. (**d**) SEM-BSE image (1 of 1383 collected) of a cross section through the vesicle showing typical morphology of the vesicle “wall” area, including balls of pyrite nanograins (arrow) in a silica matrix. (**e**) SEM-BSE image looking through a void space within the sample toward a number of vesicular compartments (red arrows) that can be directly compared to those seen in SEM images of modern pumice (see, *e.g.,* Fig. 1 of Deniz, 2012). (**f**–**g**) 3D model of the pyritic component of the reconstructed vesicle viewed from two different orientations emphasizing the spheres and semispheres of nanopyrite that comprise the wall region.

**FIG. 9.**
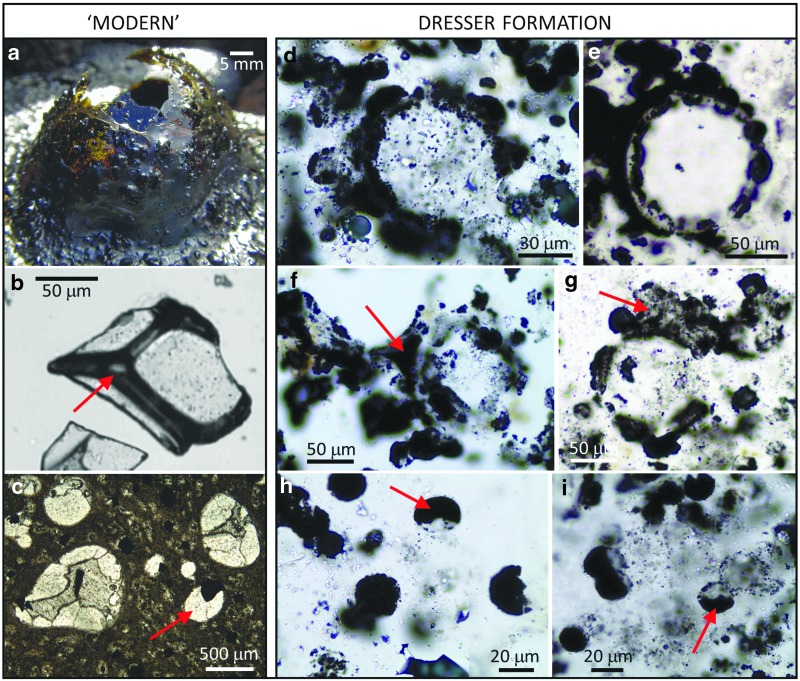
Further comparison of microstructures found within “modern” volcanic glass and those found in the Dresser Formation. (**a**) Large Kilauea lava bubble with multiple smaller bubbles on the surface; image courtesy of USGS/Hawaiian Volcano Observatory. (**b**) Fragment of 74 ka rhyolitic volcanic glass from the South China Sea showing distinct triple junction between adjacent vesicles; image reproduced with permission from Buhring *et al.* (2000). (**c**) Vesicular basalt with pyrite partially infilling the vesicles (*e.g.,* arrow), from Leg 193 of the Ocean Drilling Program; image reproduced with permission from Binns *et al.* (2002). (**d**–**e**) Examples of large vesicles with smaller “bubbles” at their margins from the Dresser Formation (compare with (a)). (**f**–**g**) Structures from the Dresser Formation interpreted as altered volcanic glass preserving “triple-junction” features comparable to that arrowed in (b). (**h**–**i**) Small vesicles from the Dresser Formation partially (arrowed) or entirely infilled with pyrite (compare with (c)).

**FIG. 10.**
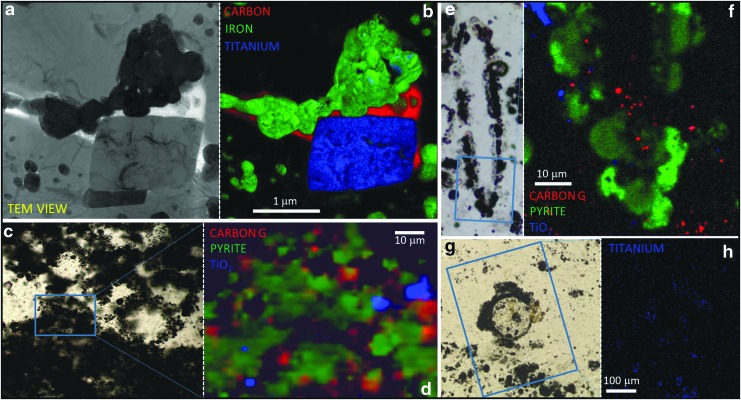
Titanium-rich minerals associated with the Dresser Formation vesicular structures. (**a**) Bright-field TEM image of part of a vesicular “wall” region, from a lenticular microstructure. (**b**) Three-color overlay EDS map of the region shown in (a) highlighting the association of pyrite (represented by green iron map), organic material (represented by red carbon map), and titanite (represented by blue titanium map); black region in this map is quartz. (**c**) Transmitted-light thin section photomicrograph of tufted lamina associated with vesicular structures. (**d**) Three-color overlay Raman phase map of the boxed region in (c) showing association of TiO_2_ (blue) with pyrite (green) and organic material (red) in the lamina; black region in this map is quartz. (**e**) Transmitted-light thin section photomicrograph of a lenticular vesicle. (**f**) Three-color overlay Raman phase map of the boxed region in (e) showing association of TiO_2_ (blue) with pyrite (green) and organic material (red) in part of the vesicle; black region in this map is quartz. (**g**) Transmitted light thin section photomicrograph of a large vesicular structure. (**h**) SEM-EDS map of titanium (blue) from the boxed region in (g) showing minor enrichment in the vesicle.

Using light microscopy and Raman spectroscopy, we observed that most vesicle walls appeared to be composed of pyrite (Figs. 4d–4o, 5a, 5c–5d). However, on closer examination, using scanning electron microscopy (SEM), transmission electron microscopy (TEM), and NanoSIMS, we could see that the vesicle walls in fact comprised over 50% quartz plus hundreds to thousands of nanograins of pyrite within this quartz matrix (Figs. 6–8). There is no continuous wall, rather a *wall region* defined by the presence of these pyrite nanograins. In some cases, where there are aggregations of multiple vesicles, these wall regions can attain thicknesses of over 100 μm and coalesce to join up adjacent vesicles (*e.g.,* Fig. 2c). In other cases, glass alteration preserves a “triple-junction” structure (Fig. 9f). Titanium-rich minerals (*e.g.,* anatase and titanite) are occasionally found associated with the wall regions of the vesicles as well as in the tufted laminae (Fig. 10).

Vesicles can be infilled in a variety of ways. Many vesicles are almost entirely quartz-filled (*e.g.,* Fig. 4h), some have alternating layers of quartz plus the same nanopyrite/quartz mixture that defines the wall regions (Fig. 4o), some have partial linings of organic particles (Fig. 10f), some have sparsely distributed organics throughout (Fig. 5c, 7b), some are entirely filled with dense balls of a mixture of nanocrystalline pyrite and quartz (Fig. 9h–9i), while others have seemingly remained partially empty (now holes in the thin section; Fig. 4m, arrowed). A minor proportion of vesicles, including large spheroidal, eye-shaped, and lenticular morphotypes, have large quantities of kerogenous carbon within or lining their wall zones, accompanied by only relatively small amounts of pyrite (Fig. 5b).

## 4. Discussion

### 4.1. Comparisons with microfossils

The Dresser vesicular microstructures exhibit a number of features that could lead to a favorable comparison to indigenous microfossils. The vesicles are abundant in each thin section and frequently occur in clusters reminiscent of microbial behavior. Many have spheroidal, elliptical, and lenticular morphologies that are closely comparable to some previously reported Archean microfossils (*e.g.,* Walsh, 1992; Sugitani *et al.,* 2007, 2010; Wacey *et al.,* 2011). Their sizes (∼10–250 μm) lie in the range of extant prokaryotes and of reported Archean microfossils, although Archean microfossils as large as 250 μm are very rare (Javaux *et al.,* 2010).

Many vesicles are associated with organic material that has a Raman signal consistent with disordered kerogen. In some cases, this organic material is concentrated in narrow regions in the vicinity of the vesicle wall (Fig. 5b). The organic material is also of a thermal maturity consistent with both the metamorphic grade of the Dresser Formation rocks and with previous reports of organic carbon in these samples (Noffke *et al.,* 2013), indicating that it is not a younger contaminant (see also Supplementary Figs. S1 and S2 for data quality control protocols that rule out laboratory contamination). In addition, the vesicles occur in sedimentary rock samples that contain both macroscopic and microscopic microbially induced sedimentary structures, as previously reported by Noffke *et al.* (2013), and potential sulfur isotope evidence for metabolic cycling (Ueno *et al.,* 2008; Shen *et al.,* 2009; Wacey *et al.,* 2015). Hence, the context of the samples is plausible for life, and evidence for life has been previously reported from this environment.

On closer examination, however, the vesicles fail a number of accepted biogenicity criteria. For example, the vesicles have thick (frequently 5–100 μm), dispersed wall zones instead of thin continuous to semicontinuous walls as demonstrated when using identical electron microscopy protocols for *bona fide* Precambrian microfossils (*e.g.,* Wacey *et al.,* 2012, 2014). *Bona fide* Precambrian cell walls can be of rather variable thickness; for example, carbonaceous walls of *Huroniospora* sp. from the 1.9 Ga Gunflint Chert range from *c.* 100 nm to 1 μm in thickness (Wacey *et al.,* 2012, 2013; Lepot *et al.,* 2017). Pyrite crystal overgrowths may locally further increase cell wall thickness during pyritization, with pyritized *Huroniospora* occasionally attaining thicknesses of 2–5 μm (Wacey *et al.,* 2013). However, this is still an order of magnitude thinner than many of the Dresser vesicles studied here (Figs. 4 and 9). The ultrastructure of the pyrite that replaces *bona fide* cells is also significantly different to that seen for the Dresser vesicles. In the Gunflint Formation, for example, the pyrite closely replicates the morphology of the original cell wall, forming a continuous ring and incorporating nanograins of quartz within pores in the pyrite (Wacey *et al.,* 2013). The Dresser wall zones, however, show the opposite pattern, with quartz seemingly incorporating nanograins of pyrite (Figs. 6–8).

Many vesicle wall zones comprise a number of linked semispheres or spheres (Figs. 8, 9d–9e), again mineralized by a mixture of quartz plus nanograins of pyrite. These spheroidal structures are rather variable in size and can be up to ∼20 μm in diameter (Fig. 9d). *Bona fide* cells can be fossilized via spheroidal growth of quartz; for example, it has been shown that silica nanospheres can nucleate and grow within cell and sheath walls in modern hot-spring environments (Schultze-Lam *et al.,* 1995), and this type of mineralization has been used as an analogue for the fossilization of microorganisms in ancient silica-rich oceans (Wacey *et al.,* 2012). However, such silica spheres are generally <500 nm in diameter and do not incorporate nanograins of pyrite, so they are not analogous to the much larger mixed-mineralogy Dresser wall spheres reported here. It is also notable that the linked spheres comprising the walls of some of the larger vesicles (*e.g.,* Fig. 9e) are essentially identical in morphology to the multitude of smaller vesicles found throughout nearby regions of the thin sections (Fig. 9h–9i), and both large and small vesicles can display the same coalescing morphology, strongly suggesting all have a common genesis.

Organic carbon is only rarely found within the wall region of Dresser vesicles. When it does occur in the wall region, it does not form any sort of cohesive structure reminiscent of a cell wall. In carbon-rich wall zones, organic carbon takes on a granular appearance and is dotted throughout the *c.* 2–10 μm width of the wall zone (Fig. 5b), while in pyrite-rich wall zones organic carbon occurs rather randomly, associated with only a small proportion of the pyrite nanograins (Figs. 6e, 7a). More commonly, organic material occurs inside the vesicles, either as a partial narrow lining (Fig. 10f) or scattered in low concentrations throughout the vesicle (Figs. 5c, 7b). Many vesicles are not associated with organic material (Fig. 5a). The majority of the organic material in the thin sections actually occurs outside the vesicles, in dense pyritized laminae (Noffke *et al.,* 2013) that appear to wrap around or envelop multiple vesicles (Fig. 2a).

The grouping and coalescence of vesicles is also inconsistent with a biological origin. Pairs, triplets, and larger groups of spheroidal vesicles often contain individual members of very different sizes (Fig. 4i, 4k, 4m–4n), inconsistent with, for example, newly divided cells or cell colonies (*e.g.,* Knoll, 2015). Similarly, adjacent lenticular vesicles can also be of significantly different sizes and have significantly different length/width ratios (Fig. 4d–4e). The common joining points between groups of vesicles are also inconsistent with an origin as cells; for example, pyritized “triple-junctions” can be several tens of micrometers across and form densely pyritized triangles with concave sides (Fig. 9f–9g, arrows).

The size frequency distribution (Fig. 3a) is unlike that of a typical biological assemblage, with no examples of vesicles in the <10 μm size category, a size which corresponds to, by far, the largest proportion of prokaryotic cells (Whitman *et al.,* 2012), a mean vesicle diameter of 57 μm, and a large standard deviation of 50 μm. This contrasts with mean microfossil diameters of 10.5 μm (st. dev. = 2.8 μm), 5.3 μm (st. dev. = 2.5 μm), and 9.3 μm (st. dev. = 1.9 μm) for cells from the ∼3.4 Ga Strelley Pool, ∼1.9 Ga Gunflint, and ∼0.9 Ga Bitter Springs Formations, respectively (Schopf, 1976; Wacey *et al.,* 2011). Finally, many of the vesicles in the 10–30 μm size range are partly or entirely filled with pyrite, so that they now resemble solid spheres or semispheres (*e.g.,* Fig. 9h–9i). Pyritization of Precambrian microfossils typically does not result in balls of pyrite; even very small (*c.* 1 μm diameter) bacteria tend to retain a hollow center (later silica filled) when observed at high spatial resolution (*e.g.,* Fig. 3 of Wacey *et al.,* 2013). The distribution of pyrite in the Dresser spheres is more consistent with the infilling of spheroidal spaces within a substrate rather than the pyritization of biological cells.

### 4.2. Comparisons with vesicular volcanic sediment

For the reasons outlined above, the Dresser vesicles cannot be interpreted as microfossils, so some other mechanism of formation is required. The Dresser Formation has been interpreted as being deposited within an active volcanic caldera (Van Kranendonk *et al.,* 2008; Djokic *et al.,* 2017), so a logical explanation for these microstructures might involve comparison with vesicular volcanic rocks.

Volcanic vesicular fabrics may commonly be heavily modified or destroyed by burial compaction and diagenesis (Branney and Sparks, 1990). However, a number of instances of vesicular pyroclastic rocks have previously been reported from the Pilbara Craton (Buick and Dunlop, 1990; Westall *et al.,* 2006; Van Kranendonk *et al.,* 2008; Brasier *et al.,* 2013). In these cases, vesicular textures appear to have been preserved by very early diagenetic silicification (*e.g.,* Brasier *et al.,* 2013). The same is likely true of the Dresser Formation, with multiple phases of hydrothermally driven silicification and pyritization demonstrated to have been occurring contemporaneously with explosive volcanic activity and sediment deposition (Van Kranendonk *et al.,* 2008). Subsequently these rocks have only experienced low-grade metamorphism (maximum of lower greenschist facies) and very little strain (Van Kranendonk *et al.,* 2007), permitting preservation of remarkable vesicular textures.

Comparisons of Dresser Formation vesicle morphology with modern volcanogenic textures are striking (Figs. 2, 4, and 9). A number of regions of the Dresser thin sections contain linked spheroids or partial spheroids that strongly resemble reworked fragments of devitrified vesicular volcanic glass (compare modern equivalent in Fig. 2b with the Dresser structures in Fig. 2c–2d). Each type of vesicle shape observed in the Dresser Formation, from spheroidal to eye-shaped to highly lenticular (Fig. 4d–4o), can be replicated in thin sections of modern vesicular volcanic glass (Fig. 4a–4c). Likewise, the log-normal vesicle size frequency distribution (Fig. 3a), pattern of cumulative vesicle size distribution (Fig. 3b), and general range of vesicle sizes are also consistent with observations of modern scoria samples (*e.g.,* Mangan and Cashman, 1996; Herd and Pinkerton, 1997; Rust and Cashman, 2011).

Some of the more unusual features of the Dresser samples, such as examples of one or more smaller vesicles lying within a larger vesicle (Fig. 4g), can also be seen in modern volcanic glass (arrows in Fig. 2a). The grouping patterns of Dresser vesicles (Fig. 4i) plus the concavo-convex relationships of shared vesicle walls (Fig. 4l–4m) are also replicated in modern vesicular volcanic textures (Fig. 4b [ringed area], 4c). The intriguing Dresser texture of multiple linked semispheres or spheres making up the wall zone of larger vesicles (Fig. 9d–9e) is analogous to a bubble wall texture found in modern lava flows (Fig. 9a), although it is at somewhat of a smaller scale. An alternative explanation for these linked pyritized “wall spheres” could be as a result of granular palagonitization of volcanic glass (*cf.* Furnes *et al.,* 2007). In this scenario, pyrite could replace the early rounded palagonite granules, while the remainder of the vesicle or glass shard is silicified. Remnants of concave triangular junctions between triplets of Dresser vesicles (Fig. 9f–9g) are analogous to those seen in modern volcanic glass fragments and diagnostic of bubble coalescence (Fig. 9b). Patterns of pyrite partially or completely infilling modern volcanic vesicles (Fig. 9c) replicate those seen for many of the smaller Dresser vesicles (Fig. 9h–9i). Primary sulfide coatings have been reported from vesicles in mafic magmas from a range of submarine settings including fresh submarine pillow basalts and seamount lavas (*e.g.,* Moore and Calk, 1971; Ackermand *et al.,* 1998).

The original mineralogy of the Dresser volcanic clasts has been almost entirely replaced by silica and pyrite, which hinders firm chemical identification of the original magmatic compositions, though the vesicular morphology has been fortuitously preserved in places by early pyritization. Traditional devitrification products of clay minerals and zeolites have also been replaced by silica. Titanium-rich minerals are observed within a small proportion of the vesicles and in some parts of the tufted laminae (Fig. 10). This is consistent with our volcanogenic interpretation but does little to determine the original magma composition. Non-uniform distribution of the vesicular microtextures suggests that the primary deposits may have been a stratified tephra sequence (perhaps vitric ash or vesicular scoria clasts) deposited into a shallow caldera lake. The vesicular textures that are preserved are more consistent with the bubble textures observed in fluidal mafic clasts (scoria), rather than the interconnected vesicle textures associated with silicic pumice (*e.g.,* Heiken and Wohletz, 1985; Rust and Cashman, 2011). Variations in extent of silicification, and pyrite content, may reflect variability in the original composition of the units, perhaps indicative of a sequence of interbedded silicic and mafic tephras. Mafic glasses would certainly have both elevated Fe and S contents, compared to silicic glasses.

There is undoubtedly an association of organic material with some of the Dresser vesicles (Figs. 5–7 and 10). Potential biofilms colonizing volcanic sediments have been reported previously from early Archean rocks of the Pilbara (3.45 Ga Kitty's Gap Chert, Westall *et al.,* 2006, 2011; 3.46 Ga Apex Basalt, Brasier *et al.,* 2013), and it has been suggested that volcanoclastic grains provided bioessential elements for chemolithotrophic biofilms, while redox reactions at their surface could provide an energy source for biological metabolism (Westall *et al.,* 2006, 2011; Brasier *et al.,* 2011). This is supported by modern experiments and observations showing that biofilms have an affinity for volcanic glass (Thorseth *et al.,* 1992; Bryan *et al.,* 2004). Previous work on this unit in the Dresser Formation has revealed several microbially induced sedimentary structures, including the tufted organic-rich laminae within these very same thin sections (Noffke *et al.,* 2013). Microbial biofilms appear to have colonized the tephra sporadically, perhaps dependent on the rate of rain-down of the ash, with colonization only in quiescent times.

### 4.3. Could some previously reported Archean microfossils be vesicular volcanic glass?

The data presented above illustrate that microstructures within vesicular volcanic glass (*e.g.,* spheres, ellipsoids, concentric spheroids, and lenses) can mimic some of the morphologies of previously reported Archean microfossils. Hence, these new data have a twofold application in the early life and astrobiology fields: firstly, they provide an alternative abiogenic hypothesis that can be tested against biogenic scenarios for future potential microfossil discoveries on the early Earth or other planets; secondly, they can be used in potential re-evaluations of previous reports of Archean microfossils. It is, of course, beyond the scope of this contribution to speculate on the origin of every Archean spheroidal or lenticular microstructure that has been claimed to be a microfossil. Unfortunately, many of these are poorly illustrated, lack geological context, and may never be analyzed with modern high-resolution techniques (see review in Wacey, 2009).

Of the Archean microfossils that are well characterized, one morphotype is particularly worthy of discussion in light of the new data presented above. Large spindle-shaped microfossils (later referred to as lenticular or flanged lenticular microfossils) have been described from the ∼3.4 Ga Kromberg Formation of South Africa (Walsh, 1992) and from the ∼3.4 Ga Strelley Pool Formation and 3.0 Ga Farrel Quartzite of Western Australia (Sugitani *et al.,* 2007, 2009, 2010, 2013, 2015a, 2015b; Grey and Sugitani, 2009; Lepot *et al.,* 2013). These microfossils seem somewhat out of place in an evolutionary context with no extant prokaryote analogues, instead being most analogous to the extant eukaryote taxon *Pterosperma,* whose previous fossil record is restricted to much younger rocks, with the earliest evidence at ∼1.3–0.9 Ga in the form of *Pterospermopsis* (Schopf and Klein, 1992) and *Pterospermella* (Samuelsson *et al.,* 1999).

The geological settings of the Kromberg, Strelley Pool, and Farrel Quartzite fossils are consistent with potential reworking of vesicular volcanics into chert sediments. For example, at least one of the Kromberg Formation cherts has been interpreted as silicified volcanic ash, while others cap volcanic flows and volcanoclastic deposits (Lowe and Knauth, 1977; Walsh, 1992). Clasts of vesicular ash have been found in the Strelley Pool Formation (Wacey *et al.,* 2017), and silicified volcanoclastics occur within a few millimeters of the black chert from which the Farrel Quartzite lenticular microfossils are described (Sugitani *et al.,* 2007).

**FIG. 11.**
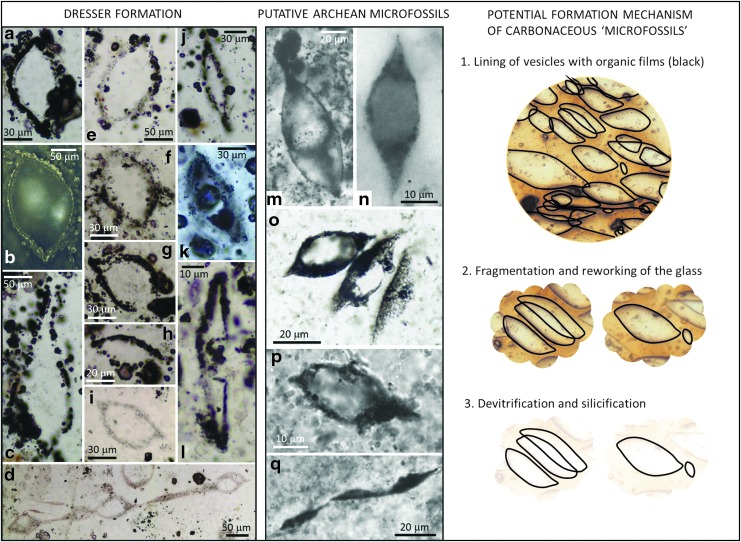
Comparison of Dresser Formation lenticular microstructures ((**a**–**l**); see also Fig. 4d–4h) with spindle-shaped and lenticular putative microfossils previously described from the ∼3.4 Ga Kromberg Formation (**m**–**n**), ∼3.0 Ga Farrel Quartzite (**o**), and ∼3.4 Ga Strelley Pool Formation (**p**–**q**). Images (m–n) reproduced with permission from Walsh (1992); image (o) reproduced with permission from Sugitani *et al.* (2007); image (p) reproduced with permission from Sugitani *et al.* (2010); and image (q) reproduced with permission from Sugitani *et al.* (2013). A potential mechanism for the creation of carbonaceous microfossil-like structures from vesicular volcanic glass is shown on the right.

The spindle/lenticular microfossils range from about 10 to 135 μm in length (Walsh, 1992; Sugitani *et al.,* 2007, 2009, 2010, 2013, 2015a; Grey and Sugitani, 2009; Lepot *et al.,* 2013; Fig. 11m–11q). In common with the Dresser microstructures described herein, individual specimens <10 μm in diameter (*i.e.,* typical size of prokaryotes) are not present. In many cases, their walls are granular and appear to be up to 15 μm thick (Walsh, 1992), which is incompatible with cell walls but closely comparable to our Dresser Formation vesicles (*e.g.,* Fig. 5b). Illustrations of some spindles in the work of Walsh (1992) show one or more spheroidal interior bodies that are closely comparable to the vesicle-within-vesicle microstructures shown in both modern vesicular glass and the Dresser Formation (Figs. 4 and 11).

Where groups of spindles are illustrated, a high proportion (*e.g.,* 62 colonies out of 80 from Sugitani *et al.,* 2013) have their long axes aligned; notably, some large spindles are associated with (or potentially attached to) one or more smaller spindles or spheres (Fig. 11m; Walsh, 1992). These features are closely comparable to our aligned Dresser vesicles of differing sizes (*e.g.,* Figs. 4e and 11d) and could be interpreted as the coalescence of spindle-shaped vesicles in ash shards. Not all previously described lenticular fossils are hollow (silica-filled); many have internal granular carbonaceous material and/or sulfides mixed with silica (Sugitani *et al.,* 2010, 2013). Likewise, some lenticular fossils lack a recognizable cell wall (Sugitani *et al.,* 2013), so they could be interpreted as void spaces filled with silica, carbon, and sulfides. Finally, the size frequency distribution of spindles shows a rather large mean and standard deviation of spindle diameters (*e.g.,* Walsh, 1992), much like our Dresser structures herein.

However, detailed analytical work by Sugitani and colleagues on the Farrel Quartzite and the Strelley Pool Formation lenticular microfossils has revealed various features that are more difficult to reconcile with a volcanogenic origin. Firstly, the lenticular structures have been described both from thin sections and from acid macerates from both the Farrel Quartzite (Grey and Sugitani, 2009) and the Strelley Pool Formation (Sugitani *et al.,* 2015a). It is unknown whether quantities of organic material that line volcanogenic vesicles would survive such an acid maceration process intact; simple aggregations of carbonaceous particles would likely disintegrate during preparation (Grey and Sugitani, 2009), but more continuous organic films (or even biofilms) colonizing volcanic vesicles may survive. It is also possible that the spindles observed in Strelley Pool, Farrel, and Kromberg thin sections are different entities to the lenticular fossils with disc-shaped flanges found in Strelley Pool and Farrel palynological extracts. If so, then the flanged microstructures are likely the best candidates for microfossils, but many of the nonflanged spindles in thin sections could be better explained as volcanogenic vesicles. Secondly, the grouping of some of the lenticular structures does not seem compatible with a volcanogenic origin. Although clusters of lenticular structures frequently have their long axes aligned (in common with volcanogenic examples), the long axes of members of several colonies are rather randomly orientated (*e.g.,* Fig. 13 of Sugitani *et al.,* 2007). Thirdly, features such as tapering carbonaceous equatorial flanges (*e.g.,* Sugitani *et al.,* 2009) and potential thin, torn, or folded walls (Sugitani *et al.,* 2007) are much more difficult to explain in a volcanogenic scenario than in a cellular scenario. Fourthly, there are δ^13^C heterogeneities within the microstructures that appear to be texture-specific (Lepot *et al.,* 2013). These heterogeneities were used to support a biological origin for the spindles and other Strelley Pool microfossils, but the data do not completely rule out the spindles forming from two (or more) generations of organic material with distinct isotopic compositions inherited from distinct organic precursors. That said, such heterogeneities would be unlikely if the spindles were volcanic vesicles lined by simple organic films unless the organics came from multiple sources, but could more easily occur if they were lined by biofilms. Experiments into the colonization of vesicular volcanic sediments by prebiotic organic films, and by biofilms, are now needed to firmly exclude such scenarios.

Large ellipsoids (up to ∼80 μm in diameter) also occur as a minor component of a suite of microfossils described from the basal sandstone member of the Strelley Pool Formation (see Figs. 1a–1b and 4a–4b of Wacey *et al.,* 2011). These have walls that are somewhat granular in appearance, and at least two examples have been shown to have a significant component of pyrite mixed with discontinuous kerogenous carbon within their walls (Wacey *et al.,* 2011). In light of the data presented above, plus the recent discovery of similar tephra within the Strelley Pool Formation itself (Wacey *et al.,* 2017), a volcanogenic origin for these large ellipsoids as well as other morphotypes within this assemblage must now be tested.

## 5. Conclusion

Herein, we have analyzed a new suite of spheroidal to lenticular microstructures from one of the oldest and best preserved volcano-sedimentary rock units on Earth, the ∼3.48 billion-year-old Dresser Formation, Pilbara Craton, Western Australia. Although these microstructures superficially resemble some types of cellular microfossils, correlative microscopy shows that they are most parsimoniously interpreted as pyritized and silicified fragments of vesicular volcanic glass. These types of microstructures represent a new type of pseudo-fossil that must be considered in future assessment of putative signs of primitive cellular life, either on Earth or elsewhere in the Universe. Given the widespread volcanic activity on the young Earth, and the propensity for such microstructures to adsorb carbon onto their surfaces, these pseudo-fossils may be particularly problematical for early or extraterrestrial life studies. Furthermore, colonization of volcanic habitats by primitive biofilms and reworking of volcanic sediment into environments already inhabited by biology may blur the boundary between abiotic vesicles and biological cells. Hence, nanoscale morphological and chemical analyses of vesicle wall structure will likely be necessary going forward in order to firmly differentiate between such pseudo-fossils and potential biological remains.

## Supplementary Material

Supplemental data

Supplemental data

## Abbreviations Used

CBS: concentric backscatter detector
CMCA: Centre for Microscopy Characterisation and Analysis
FIB: focused ion beam
NanoSIMS: nanoscale secondary ion mass spectrometer, nanoscale secondary ion mass spectrometry
ROI: region of interest
SEM: scanning electron microscope, scanning electron microscopy
TEM: transmission electron microscope, transmission electron microscopy
UWA: The University of Western Australia
