# Supplementary material for: Volcanogenic Pseudo-Fossils from the ∼3.48 Ga Dresser Formation, Pilbara, Western Australia
Source: Astrobiology. 2018 May 1;18(5):539–55. doi: 10.1089/ast.2017.1734 (PMC5963881; doi:10.1089/ast.2017.1734)
Supplement: Supplemental data [file Supp_Fig1.pdf]

Supplementary Data

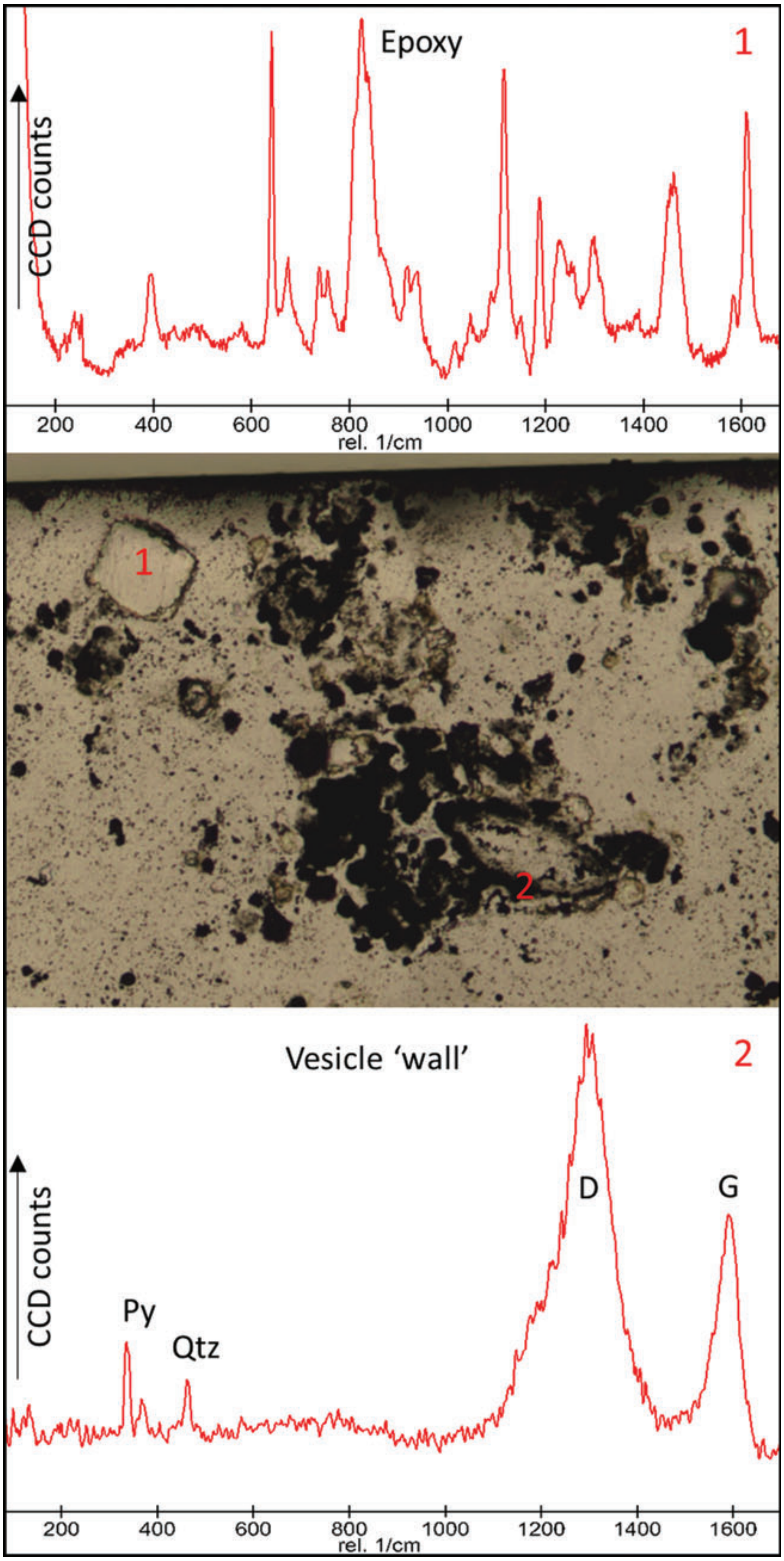

SUPPLEMENTARY FIG. S1. Identification of potential contamination of thin section surfaces by epoxy resin. The central photomicrograph shows a region near to the edge of one Dresser Formation thin section highlighting a hole filled with epoxy (1) and a vesicular microstructure (2). The lower Raman spectrum from the vesicular microstructure (2), taken from within the NanoSIMS analysis area shown in Fig. 7a, shows the distinctive carbon D and carbon G bands characteristic of Archean kerogen, plus minor quartz and pyrite peaks from the mineral matrix. In contrast, the upper Raman spectrum from the epoxy (1) shows a wide range of bands indicative of more complex modern organic material. This epoxy signal was not encountered in any of the regions analyzed by NanoSIMS.
