# Supplementary material for: Volcanogenic Pseudo-Fossils from the ∼3.48 Ga Dresser Formation, Pilbara, Western Australia
Source: Astrobiology. 2018 May 1;18(5):539–55. doi: 10.1089/ast.2017.1734 (PMC5963881; doi:10.1089/ast.2017.1734)
Supplement: Supplemental data [file Supp_Fig2.pdf]

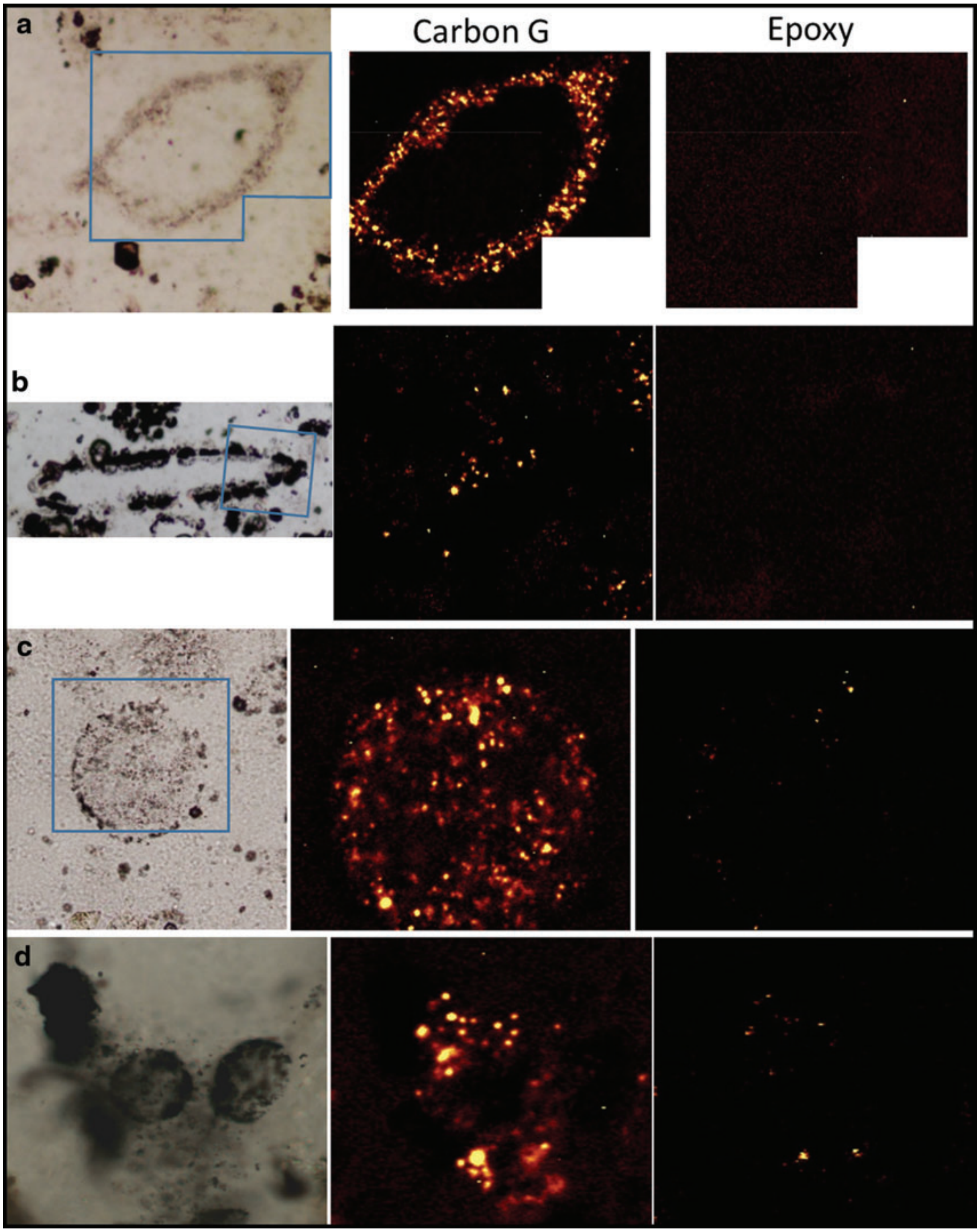

SUPPLEMENTARY FIG. S2. Raman mapping of potential contamination of thin sections by epoxy resin. Rows (**a–d**) each show thin section photomicrographs, plus corresponding carbon G ($\sim 1600\,cm^{-1}$ Raman band) and epoxy ($\sim 830\,cm^{-1}$ and confirmed by $650\,cm^{-1}$ Raman band) Raman maps for microstructures analyzed in Figs. 5 and 7. In each case the epoxy map is either featureless or contains only a handful of bright pixels. This indicates that the microstructures we analyzed contain little or no epoxy contamination, and the patterns of carbon distribution observed are indeed indigenous to the microstructures.
